# Modeling Floor Effects in Standardized Vocabulary Test Scores in a Sample of Low SES Hispanic Preschool Children under the Multilevel Structural Equation Modeling Framework

**DOI:** 10.3389/fpsyg.2017.02146

**Published:** 2017-12-12

**Authors:** Leina Zhu, Jorge Gonzalez

**Affiliations:** Psychological, Health & Learning Sciences, University of Houston, Houston, TX, United States

**Keywords:** ethnically and culturally diverse children, standardized vocabulary tests (the PPVT-4, the EVT-2), floor effects, intervention effects

## Abstract

Researchers and practitioners often use standardized vocabulary tests such as the Peabody Picture Vocabulary Test-4 (PPVT-4; Dunn and Dunn, 2007) and its companion, the Expressive Vocabulary Test-2 (EVT-2; Williams, 2007), to assess English vocabulary skills as an indicator of children's school readiness. Despite their psychometric excellence in the norm sample, issues arise when standardized vocabulary tests are used to asses children from culturally, linguistically and ethnically diverse backgrounds (e.g., Spanish-speaking English language learners) or delayed in some manner. One of the biggest challenges is establishing the appropriateness of these measures with non-English or non-standard English speaking children as often they score one to two standard deviations below expected levels (e.g., Lonigan et al., 2013). This study re-examines the issues in analyzing the PPVT-4 and EVT-2 scores in a sample of 4-to-5-year-old low SES Hispanic preschool children who were part of a larger randomized clinical trial on the effects of a supplemental English shared-reading vocabulary curriculum (Pollard-Durodola et al., 2016). It was found that data exhibited strong floor effects and the presence of floor effects made it difficult to differentiate the invention group and the control group on their vocabulary growth in the intervention. A simulation study is then presented under the multilevel structural equation modeling (MSEM) framework and results revealed that in regular multilevel data analysis, ignoring floor effects in the outcome variables led to biased results in parameter estimates, standard error estimates, and significance tests. Our findings suggest caution in analyzing and interpreting scores of ethnically and culturally diverse children on standardized vocabulary tests (e.g., floor effects). It is recommended appropriate analytical methods that take into account floor effects in outcome variables should be considered.

## Introduction

The Peabody Picture Vocabulary Test–IV (PPVT-4; Dunn and Dunn, 2007) and the Expressive Vocabulary Test–II (EVT-2; Williams, 2007), along with their earlier versions, are the most widely used standardized vocabulary tests in the United States and other countries, including: Slovania (e.g., Bucik and Bucik, 2003), France (e.g., Theriault-Whalen and Dunn, 1993), Japan (e.g., Ueno et al., 1991), Korea (e.g., Kim et al., 1995), Brazil (e.g., Capovilla and Capovilla, 1997), Northern Sotho (e.g., Pakendorf and Alant, 1997), and China (e.g., Ji et al., 2014). The popularity of these measures is evidenced by over 1,000 combined citations from 1960 to 2016 in PSYCHINFO alone. Nevertheless, debates and criticisms over use of these vocabulary tests with culturally and linguistically diverse populations continue unabated.

Criticisms of standardized vocabulary tests have ranged from content bias, bias in reference norms, threats to content and construct validity, to cultural bias and so forth (e.g., Stockman, 2000; Qi et al., 2003; Thomas-Tate et al., 2006; Haitana et al., 2010; Pae et al., 2012). Among the most salient criticism is the use of these tests with children from low-income, culturally, ethnically, and linguistically diverse backgrounds. Among vocabulary measures, The PPVT is among the most popular. The PPVT is a standardized measure of children's receptive vocabulary and screen for verbal abilities. The use of the PPVT is widespread including use in large scale federal funded early childhood programs including Even Start Programs and Early Reading First and use by speech-language pathologists for verbal ability evaluations. Its companion the EVT measures children's expressive vocabulary and complements the PPVT (Restrepo et al., 2006). The PPVT, in particular, has sparked much controversy over alleged inappropriateness with culturally and linguistically diverse populations (Haitana et al., 2010). Numerous studies have shown ethnically and linguistically diverse populations to score one to two standard deviations below normative expectations (e.g., Washington and Craig, 1992, 1999; Champion et al., 2003; Laing and Kamhi, 2003; Qi et al., 2003; Restrepo et al., 2006; McCabe and Champion, 2010; Terry et al., 2013; Gonzalez et al., 2015), highlighting possible bias in these tests. African-American, Hispanic and Native American populations, in particular, have been shown to score much lower on standardized vocabulary tests than do the normative samples (Thernstrom, 2002; Buly, 2005; Rock and Stenner, 2005; Thomas-Tate et al., 2006; Horton-Ikard and Ellis Weismer, 2007). African American children, for example, have been shown to score about one standard deviation below the mean scores compared to their White counterparts (e.g., Rock and Stenner, 2005; Restrepo et al., 2006). Latino preschoolers have been found to approach two or more standard deviations below normative standards (Lonigan et al., 2013; Gonzalez et al., 2015).

The suitability of the PPVT-4 and EVT-2 for use with ethnically, linguistically or culturally different populations continues debated. As highlighted in the manual, the PPVT-4 and EVT-2 were developed to measure standard American English (Dunn and Dunn, 2007; Williams, 2007)-a potential bias for non-standard English speaking or English learning populations. For example, neither the PPVT nor the EVT incorporate African American English (dialect of American English) in the test items (Qi et al., 2003; Pae et al., 2012). Researchers have also questioned the use of a predominately White middle-class American norm sample in both tests (Qi et al., 2003). The predominantly White norms of both tests have raised concerns in their use when testing cultural and ethnical diverse groups (Stockman, 2000; Thomas-Tate et al., 2006; Haitana et al., 2010).

Examining the appropriateness of standardized vocabulary tests for use with linguistically, culturally or ethnically different populations remains a high priority. Additionally, few efforts have been made to address how researchers can analyze data from non-English or non-standard English speaking children in ways that take into account possible biases in the tests. As discussed previously, culturally or ethnically different populations generally score disproportionately lower than the normative sample on standardized vocabulary tests. Many among these populations score at the lower end of the distribution of scores. In psychology and social science research, when test scores “stack” on or near the lower end of measurement scale, this phenomenon is known as “floor effects” (Hessling et al., 2004; McBee, 2010). Notable among tests that yield floor effects among ethnically and linguistically diverse populations are the PPVT-4 or the EVT-2. Researchers or others using the PPVT or EVT tests need to be aware that relative to the norming sample, scores for culturally and linguistically different populations may show right-skewed data distribution patterns or floor effects. Among the concerns with skewed data patterns is that many parametric statistical analytic strategies (e.g., *t*-test, ANOVA, and multiple regression) rely on normality assumptions. Inappropriate data analytical strategies result in distorted results and quite possibly erroneous or incorrect inferences due to violations of model assumption. Given that highly skewed distributions of floor effects, the use of conventional statistical methods assuming normality may yield distorted and quite possibly misleading results (Muthén and Asparouhov, 2010). For example, Hessling et al. (2004) point out that due to floor effects experimental and quasi-experimental intervention studies may fail to reject the null results when in fact the null hypothesis is rejected. As an example, if there is insufficient range in the measurement scale to capture and differentiate lower levels of ability or achievement, low-performing participants will tend to score in or “stack” at the low end of the scale. In such situations, the presence of floor effects renders it difficult to compare the invention group with the control group in terms of gains produced by an intervention. In sum, floor effects may distort efforts at examination of intervention effects, in particular, among diverse populations such as children from low-income, culturally, ethnically, and linguistically diverse background.

The importance of addressing floor effects in data analyses is largely undisputed. In both simulation and empirical studies researchers have demonstrated that ignoring floor effects can result in biases in parameter estimates, standard error estimates, and misleading inferences (Wang et al., 2008; Twisk and Rijmen, 2009; McBee, 2010). To address potential floor effects in data analysis, techniques to deal with the floor and similar type of data have been developed and increasingly applied in social science research (e.g., Twisk and Rijmen, 2009; McBee, 2010; Proust-Lima et al., 2011; Iachina and Iachina, 2012; Whitaker and Gordon, 2012; Keeley et al., 2013). For example, the practice of treating floor data as left-censored data and using the Tobit regression model as a correction has been a common recommendation (e.g., Cox and Oakes, 1984; Muthén, 1989, 1990; Klein and Moeschberger, 1997). The concept of floor effects is similar to left-censoring in survival analysis framework. In survival analysis, left censoring is considered to when some individuals have already experienced the event of interest before recording or observing or collecting those targeted data points (Kleinbaum and Klein, 2005). Floor effects are in similar nature. Due to a measurement range that does not adequately capture extremely low levels of ability and/or achievement, some true scores beyond the scale limits cannot be observed, similar to the left-censored data which are censored/truncated at the lower-boundary (floor threshold). While left censoring is related to the observation time, floor effects are in the context of restricted range of measurement. In Tobit regression model (also called censored regression), from treating floor data as left-censored data, Tobit regression effectively models the limitation.

Recognizing that floor effects in data analysis can lead to biased estimates, it is recommended that researchers more closely examine the distribution of scores in standardized measures administrated to diverse populations. For example, in a sample of students with special needs, Whitaker (2005, 2008, 2010, 2012) identified possible floor effects in their scores on both the Wechsler Adult Intelligence Scale (WAIS) and the Wechsler Intelligence Scale for Children (WISC). Similarly, while screening a large cohort of students for reading disabilities, Catts et al. (2009) pointed out floor effects in their scores on the Dynamic Indicators of Basic Early Literacy Skills (DIBELS), a screening instrument for identifying children at risk for reading disabilities. Many children were found to score near the lower end of the distribution (no or low risk for reading disabilities). These studies demonstrate that floor effects may occur when these measures are used with diverse groups. Nevertheless to our knowledge, no studies exist examining the impact of floor effects in administrations of the PPVT-4 or EVT-2 with children from low-income, culturally, ethnically, and linguistically diverse backgrounds. As discussed previously, numerous studies found these children often performed poorly on the PPVT-4 or EVT-2 with the vast majority of scores stacked near the lower end of the data distribution. While there is no universally accepted definition of what constitutes floor effects in tests, in some disciplines (e.g., clinical orthopedics research), floor effects are defined as when 15% (or more) of sample participants score at the lowest level of a measure's range (Lim et al., 2015). Given the predominance of low scores for cultural and ethnical diverse groups on the PPVT-4 or EVT-2, particular attention needs to be paid to the presence of floor effects in the data.

## The purpose of the study

Despite psychometric excellence of the PPVT-4 and the EVT-2, concerns arise when these tests are used to asses diverse populations who may perform substantively different from the norm sample. As noted in research, non-English or non-standard English speaking children often score one to two standard deviations below normative standards (e.g., Lonigan et al., 2013). This study focused on Mexican-American Spanish-speaking preschool dual language learners (DLL) enrolled in preschool.

The study had three aims: (a) to examine floor effects in data from a the pre-test administration of the PPVT-4 (Dunn and Dunn, 2007) and the EVT-2 (Williams, 2007) test scores in a sample of low SES Mexican-American DLL preschool children (Pollard-Durodola et al., 2016), (b) to examine the impact of floor effects on evaluating the pre- post-test performance on receptive and expressive vocabulary outcomes as measured by the PPVT-4 and the EVT-2 (Pollard-Durodola et al., 2016), and (c) to evaluate the impact of floor effects on estimating parameters, standard errors, and significant tests through Monte Carlo simulations. Different analytical approaches were compared in response to different levels of floor data in the outcome variable in the multilevel structural equation modeling (MSEM) framework, which is viewed as a more general framework to analyze multilevel data. Results discussed and appropriate statistical methods for dealing with data with floor effects were thereby suggested.

## Dealing with floor effects

In this study, we examined three methods of analyzing data from pre and post administration of the PPVT-4 and EVT-2 with potential floor effects, including the regular multilevel regression model with maximum likelihood (ML) estimation (ignoring floor effects), the robust standard error approach in multilevel model (standard error adjustment based on maximum likelihood with robust standard error estimation), and the multilevel Tobit regression model (addressing floor effects from treating the outcome variable with floor effects as left-censored variable). All these analyses are set up under the multilevel structural equation modeling (MSEM) framework given that MSEM is viewed as a more general framework for analyzing multilevel data with the flexibility to include both observed and latent variables in the model simultaneously (Muthén and Muthén, 1998-2015). Conventional linear regression assumes normality assumption. Floor effects in the dependent variable are not taken into account in the conventional linear regression analysis (Winship and Mare, 1984). The robust standard error approach and the Tobit approach, on the other hand, handle floor effects with different techniques. In the next section, the latter two approaches are presented in more detail.

### The robust standard error approach

Statistical methods often rely on certain assumptions, such as multivariate normality, homoscedasticity, or observation independency. If model assumptions are not satisfied, substantial biases would occur in parameter estimates, standard error estimates, and model evaluation. Floor effects generally occur when data distributions are highly right skewed. Given floor effects in the outcome variables, the use of linear regression is problematic due to potential violation of the multivariate normality assumption. Yuan et al. (2005), for example, demonstrated that standard error estimates and test statistics may be inconsistent due to data nonnormality (e.g., positive skewed data). Brown (2006) noted marked floor effects led to biased standard error estimates using maximum likelihood (ML). Note that in some conditions, normal theory ML produced unbiased parameter estimates though data are nonnormal, however, bias in standard error estimates cannot be overcome and possibly distorting significance testing, and in turn misleading inferences (Yuan and Bentler, 2000; Finney and DiStefano, 2006; Baraldi and Enders, 2010).

To correct for bias in standard error estimates, robust standard error approach has often been used to produce unbiased standard errors (King and Roberts, 2015). The literature identifies several ways to obtain robust standard errors, such as asymptotically distribution-free estimation (ADF; Browne, 1984) and bootstrapping (Nevitt and Hancock, 2001). Other methods include Huber/Pseudo sandwich estimator. In the M*plus* program (Muthén and Muthén, 1998-2015), there are three routines to produce “robust” standard errors, including: (1) maximum likelihood parameter estimates with robust standard errors and chi-square test statistic (MLM), (2) maximum likelihood parameter estimates with standard errors and a mean- and variance-adjusted chi-square test statistic that are robust to non-normality (MLMV), and (3) maximum likelihood parameter estimates with standard errors and chi-square test statistic robust to non-normality and observation non-independence (MLR). In this study, M*plus* was used for all analyses and illustrations.

The three estimation methods, namely, MLM, MLMV, and MLR, are all ML based robust estimators. However, standard errors produced by these ML estimators could be very divergent. In many situations, the ML parameter estimates are still consistent even data are nonnormal, but standard error estimates could be very biased. In analyzing multilevel data, MLR shows its advantage in dealing with observation dependency (Maas and Hox, 2004). In addition, MLR is also superior in handling: (1) data non-normality and (2) missing data (see Yuan and Bentler, 2000). In this study, we adopted MLR estimator in terms of handling both floor effects in data and data of multilevel structure.

### The tobit approach

Tobit regression analysis, first formulated by Tobin (1958), models linear relationships between variables when the outcome variable is either a left- or right-censored variable. In Tobit regression, scores that fall at or below some threshold are viewed to be (left) censored from below the threshold. As described previously, floor effects are potential when a large percentage of scores occurs at the low end of the measurement scale. Data with floor effects are treated as left-censored data in Tobit regression. For instance, when two low-performing students are measured with a standardized test, both students scored zero on the test, but their actual abilities may not be the same. In this case, their scores seem to be censored from the censoring point (i.e., zero), which however, fail to capture their true abilities. The standardized test, because of its restricted score range, is unable to differentiate abilities of students who score extremely low (or high) level. Scores at the extremes can be viewed as being censored or truncated. The lowest (or highest) bound is called the censoring point or threshold (Cox and Oakes, 1984). To sum, in the Tobit regression model dependent variables with floor effects are viewed as left-censored variables.

In the Tobit regression model, y^*^ represents a random latent variable and y represents a censored variable. When the data are not censored, the distributions of y^*^ and y overlap. The lowest bound is defined as “*l*” and the highest bound as “*u*”. Mathematically, the Tobit regression models are expressed as follows: (Long, 1997; Twisk and Rijmen, 2009):

(1) $$y_{i}^{*}\;=\;\beta_{0}+\beta_{1}x_{i}\prime +e_{i},e_{i}\sim N(0,\;\sigma^{2}),$$

(2) $$y_{i}=l\text{ for }y_{i}^{*}\leq l,$$

(3) $$y_{i}=y_{i}^{*}\text{ for }l<y_{i}^{*}$$

when the outcome variable is left-censored.

When the outcome variable is right-censored, expressions include

(4) $$y_{i}^{*}\;=\;\beta_{0}+\beta_{1}x_{i}\prime +e_{i},e_{i}\sim N(0,\;\sigma^{2}),$$

(5) $$y_{i}=y_{i}^{*}\text{ for }y_{i}^{*}<u,$$

(6) $$y_{i}=u\text{ for }y_{i}^{*}\geq u.$$

The discussion till now was about a simple Tobit regression model. When data are characterized by dependency among observations due to the nested or hierarchical data structure (e.g., students nested within classrooms, members nested within organizations), multilevel model (MLM) is the appropriate method (Raudenbush and Bryk, 2002; Hox et al., 2010). The following expressions (7–9) represent a typical multilevel model (i.e., random intercept model which is equivalent to a commonly used form of multilevel structural equation model (MSEM) and can be specified and analyzed by the M*plus* Type = Twolevel routine):

(7) $$\text{Level 1}:{\text{Y}}_{ij}=\beta_{0j}+\beta_{\text{1j}}{\text{X}}_{ij}+{\text{e}}_{ij},$$

(8) $$\text{Level 2}:\;\beta_{\text{0j}}=\gamma_{\text{00}}+{\text{U}}_{\text{0j}},$$

(9) $$\beta_{1j}=\gamma_{10},$$

where *i* represents the individual (i.e., *i* = 1…*n*_j_) and *j* represents the group in which the individual is nested (i.e., *j* = 1…*N*). In the level-1 model as shown in Equation (7), β_0*j*_ is the estimated average for the *j*-th group. B_1*j*_ is the slope which is a fixed effect and X_*ij*_ is the level 1 covariate. *e*_*ij*_ is the within-group random error. In the level-2 models shown in Equations (8) and (9), β_0*j*_ is the random intercept constituted by the grand mean (γ_00_) and the between-group random effect (U_0*j*_). We interpret the estimate for U_0*j*_ as the variance of the mean for each group around the grand mean. In Equation (9), given the slope is a fixed effect, γ_10_ represents the average change across all groups for the X_*ij*_ predictor.

To address floor effects in the outcome variable, the above Equations (7), (8), and (9) can be modified and the following equations represent a multilevel Tobit regression model:

(10) $$\text{Level 1}:y_{ij}^{*}=\beta_{0j}+\beta_{\text{1j}}x_{ij}\prime +{\text{e}}_{ij},$$

(11) $$y_{ij}=l\text{ for }y_{ij}^{*}\leq l,$$

(12) $$y_{ij}=y_{ij}^{*}\text{ for }l<y_{i}^{*}$$

(13) $$\text{Level 2}:\beta_{0j}=\gamma_{00}+{\text{U}}_{0j},$$

(14) $$\beta_{1j}=\gamma_{10},$$

in which the outcome variable with floor effects (*y*_*ij*_) is treated as left-censored ($y_{ij}^{*}$) in the multilevel Tobit regression model.

Next, three comparative methods, including regular multilevel regression, multilevel regression with robust standard error approach, and multilevel Tobit regression, were examined in response to floor effects in data with multilevel structure. The first method did not address the floor effects. The latter two methods addressed the floor effects differently. For the robust standard error approach, the MLR estimator was adopted to obtain robust standard errors. For the multilevel Tobit regression approach, the outcome variable was treated as a left-censored variable in which a multilevel Tobit regression was applied. Next, we presented two studies to examine the three methods. First an empirical example was presented, followed by a simulation study.

## An empirical example

Although there is no consensus, standardized vocabulary tests (e.g., the PPVT-4 and the EVT-2) may, under some circumstances, be inappropriate for use with culturally or linguistically diverse populations. Given evidence of low standardized vocabulary tests scores from some cultural and linguistically diverse groups, this study highlighted the potential issue of floor effects. In summary, the aim of this empirical example was 2-fold: (a) to establish the existence of floor effects on the PPVT-4 and the EVT-2 test scores in a sample of low SES Mexican-American preschool children who were dual language learners (DLLs) (Pollard-Durodola et al., 2016), and (b) to investigate the impact of floor effects on examining the PPVT-4 and the EVT-2 pre- to post-test scores comparison with respect to the sample's vocabulary growth (Pollard-Durodola et al., 2016).

In this example, the participants included 252 low-income Mexican-American preschool children participating in randomized clinical trial of an evidence-based shared book reading intervention in two school districts located in South Texas. In this sample, preschool children (average age was 5 years) were 92.1% economically disadvantaged, and primarily Mexican-American (98.3%). Eighty-seven percent of parents of preschoolers reported that Spanish was the primary language spoken at home while 8% reported speaking English in the home and 5% reported using both languages. All children were identified as Spanish-speaking children while learning English as a second language (Pollard-Durodola et al., 2016). Based on the student performance on the *pre*LAS® English (DeAvila and Duncan, 2000), all preschoolers were at the pre-functional and beginning level for their English language proficiency. All the preschool children were assessed using the PPVT-4 and the EVT-2 at pre- and posttests to examine the impact of the shared-reading intervention on their vocabulary growth.

Table 1 provides descriptive on standardized scores for the post-test PPVT-4 and the EVT-2. As shown in Table 1, a significant majority of participants scored in the low range, including: 93.97% on the PPVT-4 (i.e., moderately low range 33.73% + extremely low range 60.24%) and 92.01% on the EVT-2 (i.e., moderately low range 31.09% + extremely low range 60.92%). The mean score on PPVT-4 was 63.81 (*SD* = 15.42), corresponding to two standard deviations (*SD*s) below the normative mean of 100. The mean score on the EVT-2 was 55.63 (*SD* = 24.01), corresponding to three standard deviations (*SD*s) below the normative mean of 100 The standardized scores on the PPVT-4 ranged from 20 to 91, which indicated that all participants (*N* = 252) scored below the normative mean (i.e., 100). On the EVT-2, the scores ranged from 20 to 108. Only two out of 252 participants (i.e., 1%) scored above the normative mean (i.e., 100) while 99% (i.e., *n* = 250 out of 252) scored below the normative mean (i.e., 100). In summary, post-test standardized scores on the PPVT-4 and EVT-2 for the sample of low SES Mexican-American preschool children suggested evidence of floor effects.

**Table 1:** Distribution of scores and corresponding descriptive for all of the participants.

**Descriptor**	**Standard score range**	**Measures**			
		**PPVT-4 (*N* = 249)**		**EVT-2 (*N* = 238)**	
		***n***	**Percentage**	***n***	**Percentage**
Extremely high	130+	0	0	0	0
Moderately high	115–129	0	0	0	0
High average	100–114	0	0	2	1
Low average	85–99	15	6	17	7
Moderately low	70–84	84	34	74	31
Extremely low	≤69	150	60	145	61

**Figure 1:**
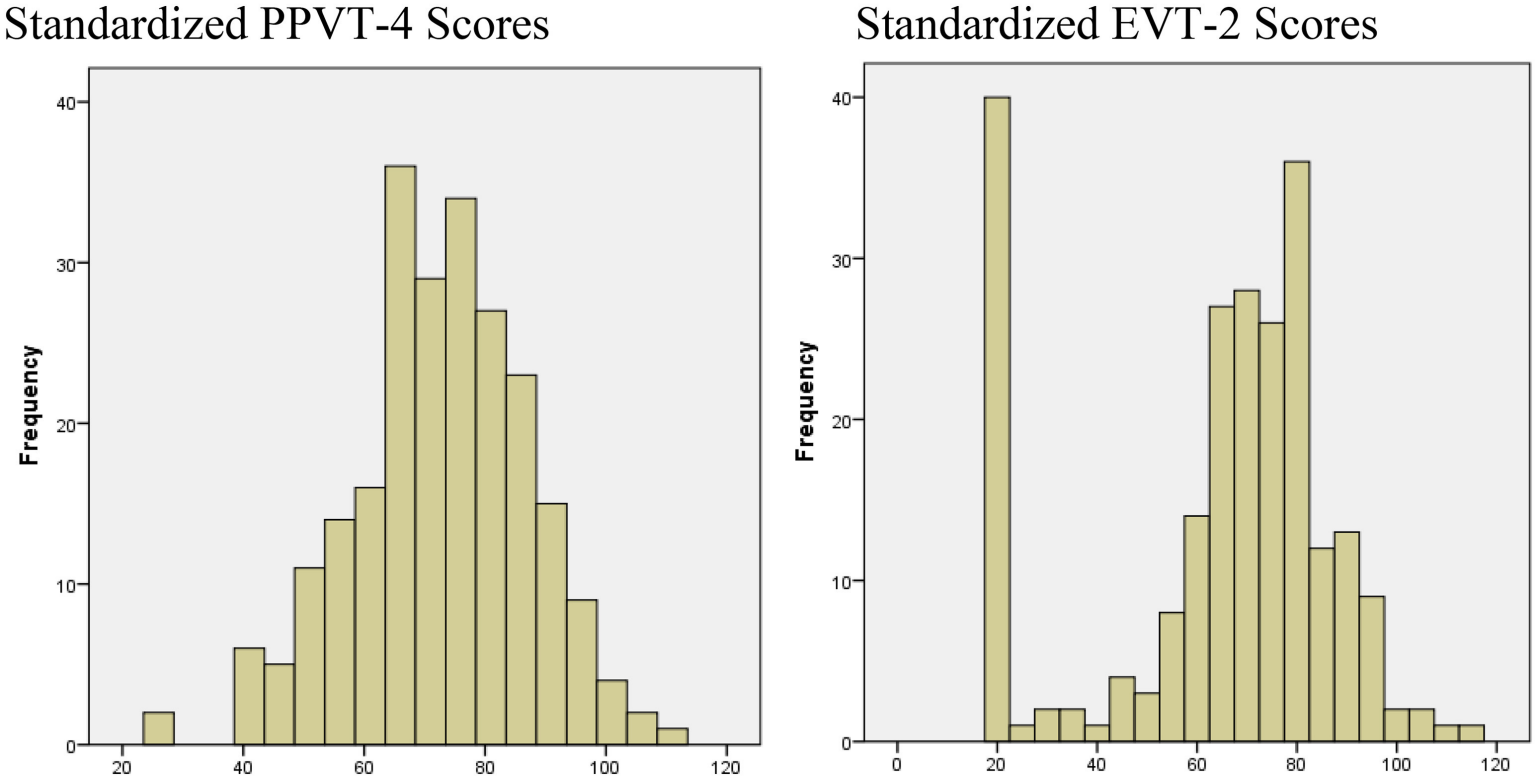
Distribution of standardized vocabulary test scores of a sample of low SES Hispanic preschool children.

Figure 1 displays the distributions of standardized PPVT-4 and EVT-2 scores of the sample of preschool children, respectively. The distributions demonstrate that a preponderance of scores fell in the low ranges, especially on the EVT-2. As noted earlier, if 15% or more of the sample scores in the lowest level of a measure range, floor effects likely exist. Regardless of the standardized scores or the raw scores, this sample of Mexican-American preschool children performed significantly lower relative to the norm sample on the PPVT-4 and the EVT-2 with the vast majority scoring on or near the low end of measurement scale.

In the second aim of this study, we explored the influence of floor effects on the pre- to post effectiveness of the shared-reading intervention on the sample of Mexican-American children. Specifically, we wanted to know whether floor effects masked vocabulary growth. As shown in Table 2, at pre-test the children scored on average two standard deviations (*SD*s) below the normative mean on the PPVT-4 and three standard deviations (*SD*s) below the normative mean on the EVT-2. The children still lagged behind at posttests on average showing one standard deviation (*SD*s) below the normative mean on the PPVT-4 and two standard deviations (*SD*s) below the normative mean on the EVT-2. When having the first glance at the post-test scores, there appeared to be no difference between the intervention and control groups on the two standardized vocabulary measures: PPVT-4: *t* = 0.53, *p* = 0.599; EVT-2: *t* = −0.30, *p* = 0.762. According to the pretest-posttest comparison, one could reasonably conclude that the intervention had been ineffective in accelerating vocabulary growth for the treatment group of children. One interpretation would be that the shared book reading intervention designed to show promise in improving children's vocabulary for diverse children was not effective. Results must, however, be interpreted in light of the staggeringly poor performance of the Mexican-American preschoolers at pretest (e.g., many children scored two to three standard deviations below monolingual vocabulary norms). Because their pre-test vocabulary performance was so low, it appears that these preschool children were unresponsive to the intervention. In this scenario analyzing performance using conventional analytic methods on the preschool children's pre- to post-test PPVT-4 and EVT-2 without adequately taking into account of the floor effects may have resulted in misleading conclusions about the effectiveness of the intervention.

**Table 2:** Pretest and posttest scores for intervention and comparison groups.

**Measure**	**Pretest**				**Posttest**			
	**Total**	**Intervention**	**Comparison**	***t***	**Total**	**Intervention**	**Comparison**	***t***
**PPVT-4**								
*N*	249	136	113	0.05, *p* = 0.957	234	129	105	0.53, *p* = 0.599
*M*	63.81	63.86	63.75		72.70	73.16	72.13	
*SD*	15.42	14.76	16.24		14.63	14.12	15.29	
**EVT-2**								
*N*	238	132	106	0.17, *p* = 0.864	232	127	105	−0.30, *p* = 0.762
*M*	55.63	55.87	55.33		64.08	63.65	64.60	
*SD*	24.01	23.59	24.64		24.01	25.09	22.75	

*PPVT-4, Peabody Picture Vocabulary Test (4th ed.); EVT-2, Expressive Vocabulary Test (2nd ed.)*.

Table 3 presents model results using the three different methods (i.e., the traditional multilevel model without addressing the floor effects, the robust standard error approach which partially addressing the floor effects, and the multilevel Tobit model which directly addressing the floor effects). An annotated input from the M*plus* program for analyzing a multilevel Tobit model was presented in Appendix A. As shown in Table 3, the results showed a mixed pattern, for example, some parameter estimates appeared to be larger when floor effects were considered (e.g., intervention) whereas some other estimates tended to be smaller (e.g., pretest, *pre*LAS® English). Specifically, for the EVT-2 outcome (with stronger floor effects compared with the PPVT-4 outcome), the approaches which accounting for floor effects yielded larger parameter estimates (e.g., gender, intervention, and years of teaching). Regarding the standard error estimates, methods addressing floor effects generally produced smaller standard errors than the traditional multilevel model which ignoring floor effects.

**Table 3:** Results of hierarchical linear model to the low SES Hispanic Latino preschool children with or without modeling floor effects.

**Parameter estimates and standard errors**	**Dependent variable**					
	**PPVT-4**			**EVT-2**		
	**Multilevel model floors ignored**	**Robust standard error**	**Multilevel model floors considered**	**Multilevel model floors ignored**	**Robust standard error**	**Multilevel model floors considered**
**FIXED EFFECTS**						
Intercept	35.97^*^	35.97^*^	36.93^*^	1.62	1.62	0.51
(*SE*)	(13.46)	(14.54)	(15.42)	(3.10)	(3.13)	(2.84)
Level-1 Pretest (γ_10_)	0.98^*^	0.98^*^	0.98^*^	0.83^*^	0.83^*^	0.82^*^
(*SE*)	(0.01)	(0.01)	(0.01)	(0.03)	(0.03)	(0.04)
Level-1 Age (γ_20_)	−0.01	−0.01	−0.01	−0.002	−0.002	−0.01
(*SE*)	(0.02)	(0.02)	(0.02)	(0.05)	(0.04)	(0.04)
Level-1 Gender^a^ (γ_30_)	−0.004	−0.004	−0.004	−0.45	−0.05	−0.03
(*SE*)	(0.02)	(0.02)	(0.02)	(0.04)	(0.04)	(0.04)
Level-1 Bilingual^b^ (γ_40_)	−0.04	−0.04	−0.04	−0.05	−0.05	−0.05
(*SE*)	(0.03)	(0.02)	(0.03)	(0.06)	(0.06)	(0.06)
Level-1 Ethnicity^c^ (γ_50_)	0.003	0.003	0.003	−0.02	−0.02	−0.02
(*SE*)	(0.02)	(0.01)	(0.01)	(0.04)	(0.04)	(0.05)
Level-1 Attendance (γ_60_)	0.02	0.02	0.02	0.01	0.01	0.01
(*SE*)	(0.02)	(0.02)	(0.02)	(0.04)	(0.04)	(0.05)
Level-1 *pre*LAS® English (γ_70_)	0.000	0.000	0.000	0.05	0.05	0.04
(*SE*)	(0.02)	(0.02)	(0.02)	(0.05)	(0.04)	(0.05)
Level-1 *pre*LAS® Spanish (γ_80_)	0.001	0.001	0.002	−0.01	−0.01	−0.01
(*SE*)	(0.02)	(0.02)	(0.02)	(0.05)	(0.04)	(0.05)
Level-2 School district^d^ (γ_01_)	−0.70	−0.70	−0.73	−0.58	−0.58^*^	−0.54
(*SE*)	(0.46)	(0.37)	(0.39)	(0.39)	(0.25)	(0.23)
***Level-2 Intervention (**γ_02_**)***	***0.24***	***0.24***	***0.25***	***0.40***	0.40^*^	0.41^*^
***(SE)***	***(0.25)***	***(0.22)***	***(0.24)***	***(0.21)***	***(0.19)***	***(0.19)***
Level-2 Teacher's primary language^e^ (γ_03_)	−0.59^*^	−0.59^*^	−0.60^*^	−0.35	−0.35	−0.31
(*SE*)	(0.28)	(0.23)	(0.24)	(0.22)	(0.23)	(0.22)
Level-2 Years of teaching (γ_04_)	−0.81^*^	−0.81^*^	−0.81^*^	−0.44	−0.44	−0.39
(*SE*)	(0.40)	(0.40)	(0.41)	(0.32)	(0.40)	(0.40)
Level-2 Years of teaching in PreK (γ_05_)	−0.22	−0.22	−0.23	−0.52	−0.52	−0.57
(*SE*)	(0.34)	(0.41)	(0.42)	(0.29)	(0.31)	(0.33)
Level-2 University reading credits (γ_06_)	1.11^*^	1.11^*^	1.14^*^	0.86^*^	0.86^*^	0.87^*^
(*SE*)	(0.40)	(0.37)	(0.39)	(0.30)	(0.34)	(0.32)
Level-2 Professional development (γ_07_)	0.03	0.03	0.06	0.63	0.63^*^	0.57
(*SE*)	(0.45)	(0.39)	(0.40)	(0.37)	(0.27)	(0.26)
**RANDOM EFFECTS**						
Level-1 Residual Variance (σ^2^)	0.06^*^	0.06^*^	0.06^*^	0.29^*^	0.29^*^	0.32^*^
(*SE*)	(0.01)	(0.01)	(0.01)	(0.04)	(0.05)	(0.05)
Level-2 Residual Variance (τ _00_)	0.23	0.23	0.19	0.46	0.46^*^	0.48^*^
(*SE*)	(0.39)	(0.34)	(0.37)	(0.25)	(0.20)	(0.20)

* *PPVT-4, Peabody Picture Vocabulary Test (4th ed.); EVT-2, Expressive Vocabulary Test (2nd ed.)*. *The significance level is set at p < 0.05 (two-tailed)*.

a *The reference group for gender is female (coded 0)*.

b *The reference group for bilingual is non-bilingual (coded 0)*.

c *The reference group for ethnicity is Native American (coded 0)*.

d *The reference group for school district is school district A (coded 0)*.

e *The reference group for teachers' primary language is English (coded 0)*.

*Bold and italic values indicated a contrast of significant effects to non-significant effects when floor effects were addressed regarding the intervention effects which is the target research interest in the empirical study*.

The most intriguing findings in Table 3 were the potential influence of floor effects on testing the intervention effects (i.e., γ_02_ in Table 3). Non-significant intervention effects were detected for the PPVT-4 and EVT-2 outcomes when using the regular multilevel regression without addressing the floor effects. Nevertheless, both robust standard error approach (partially addressing the floor effects) and Tobit regression approach (fully addressing the floor effects) yielded significant intervention effects on the EVT-2, the measure of expressive vocabulary, but non-significant intervention effects on the PPVT-4, the receptive vocabulary measure. By further examining the descriptive statistics as shown in Table 1, we found that more children scored near the lower end of the EVT-2 than on the PPVT-4. These results validated that the necessity of taking the floor effects into account when conducting the data analysis with potential floor effects. Without properly addressing the floor effects, one can result in the incorrect test of the significant intervention effect and mislead to the non-significant intervention effect conclusion.

In summary, floor effects were shown to be present in both standardized receptive and expressive vocabulary tests scores in a sample of low income Mexican-American preschool children who enrolled in a randomized clinical trial of a shared-book reading intervention (Pollard-Durodola et al., 2016). Analytical methods ignoring the floor effects (i.e., regular multilevel regression) and methods addressing the floor effects (i.e., robust standard error approach partially addressing the floor effects and Tobit regression approach fully addressing the floor effects) resulted in difference in model results. Accounting for floor effects in data analysis yielded different results (i.e., standard error estimates and significance tests), though parameter estimates did not appear to be significantly impacted. When floor effects were ignored, standard errors tended to be overestimated. On the other hand, both robust standard error and Tobit regression approaches produced smaller standard error estimates and subsequently significant results. Hence, we would like to further examine whether partially addressing the floor effects (i.e., the robust standard error approach) would be sufficient enough to obtain unbiased parameter estimates and standard errors, or only fully addressing the floor effects (i.e., Tobit regression) would result in unbiased estimates and standard errors.

## The simulation study

To further examine the impact of floor effects in multilevel data analysis, a Monte Carlo simulation study was conducted. Using the Monte Carlo routine in M*plus* version 7.31 (Muthén and Muthén, 1998-2015), data with floor effects were generated. Next, the simulated data were analyzed using the three comparative methods: (a) the maximum likelihood (ML) based multilevel regression model without addressing the floor effects, (b) the robust standard error approach (i.e., the ML based multilevel regression model with robust standard error estimator) only partially addressing the floor effects, and (c) the multilevel Tobit model which fully addressing the floor effects by defining the outcome variable as a left-censored variable.

### Data generation

Data were simulated based on a basic two-level random intercept model which was a commonly used multilevel structural equation model and could be fitted with the M*plus* Type = Twolevel routine. Floor effects in the outcome variable were considered. The population model for data generation was as follows. The fixed effects parameter vector (γ_00_, γ_10_) represented the grand mean and slope. ϕ represented the between-level variance and $\sigma_{i}^{2}$ was the within-level residual variance.

(15) $$\text{Level 1}:{\text{Y}}_{\text{ij}}=\beta_{\text{0j}}+\beta_{\text{1j}}{\text{X1}}_{\text{ij}}+{\text{e}}_{\text{ij}}$$

(16) $$\text{Level 2}:\beta_{\text{0j}}=\gamma_{00}+\gamma_{01}\text{X}2_{\text{j}}+{\text{U}}_{\text{0j}}$$

(17) $$\beta_{\text{1j}}=\gamma_{10}$$

(18) $${\text{U}}_{\text{0j}}\sim N(0,\phi )$$

(19) $${\text{e}}_{\text{ij}}\sim N(\text{0},\sigma_{\text{i}}^{2}).$$

Population parameters used to generate the data are as follows: the variances of X1 and X2 were both 1. The means of X1 and X2 were set to be zero. The within-level residual variance ϕ and the between-level residual variance $\sigma_{i}^{2}$ were set to equal 1 and 0.5, respectively. The between-level mean of Y was set to be 1. The parameter vector (γ_10_, γ_01_) was set to be (0.75, 0.50). The outcome Y_*ij*_ was simulated with different proportions of floor data, which was detailed in a later section. Sample size was 1,000. Five hundred replications were generated for each simulation condition.

**Figure 2:**
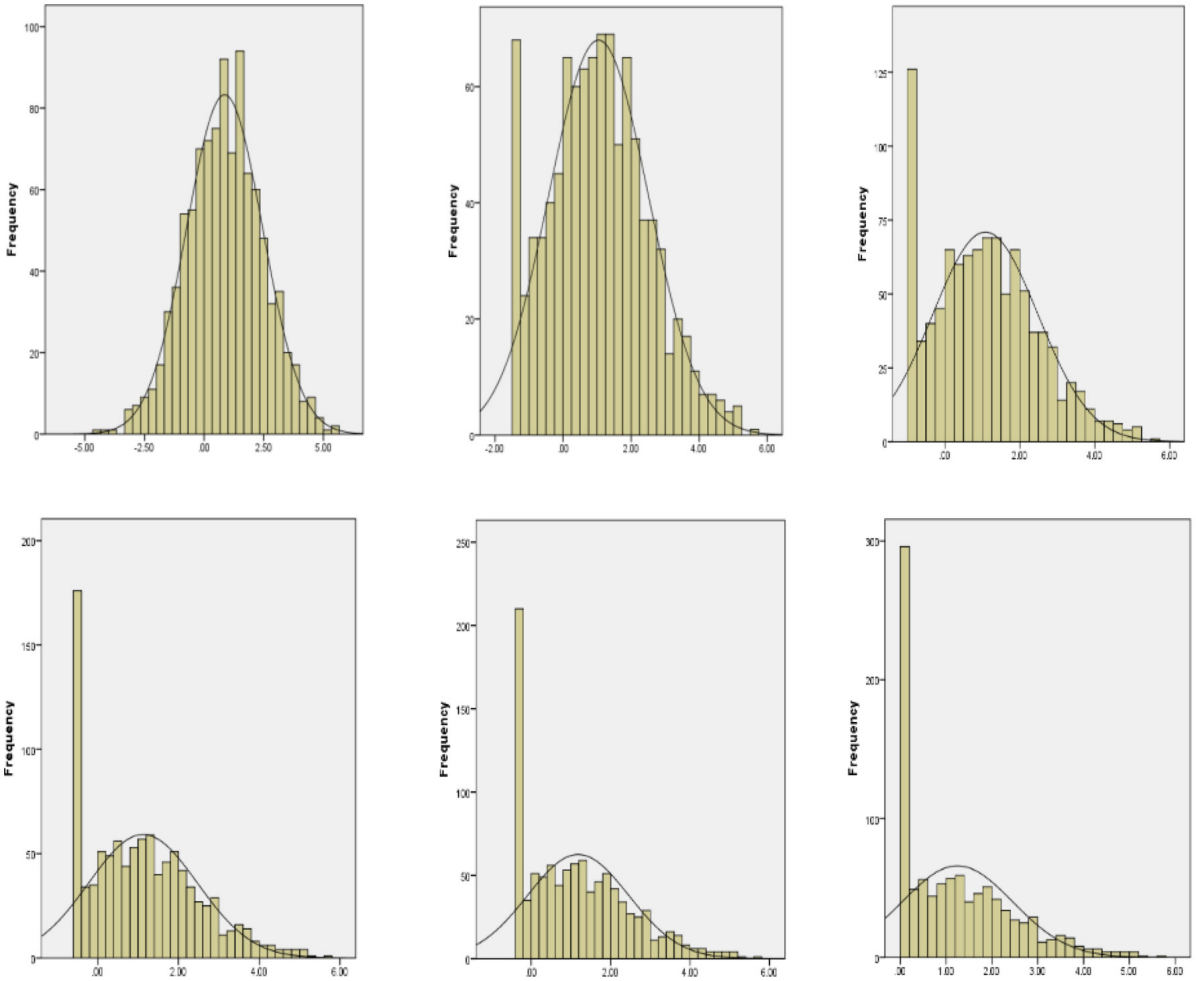
Distribution of six simulated data sets showing different proportions of floor data (from 0 to 25% floor data).

Regarding the different proportions of floor data in the outcome Y, six conditions (i.e., 0, 5, 10, 15, 20, and 25%) were considered, with 0% representing no floor effects and 25% representing the most floor effects. In the study by Wang et al. (2008), the authors used different ceiling thresholds to manipulate different ceiling proportion conditions in studying ceiling effects. In this study, we adopted their approach and varied the left-censoring points to create different proportions of floor data. The floor proportions and floor thresholds are presented in Table 4. The proportion of floor data increased as floor thresholds increased. Figure 2 displays the corresponding distributions of the six simulated data sets with different proportions of floor data in the outcome variable. In the 0% floor data condition, the data was shown to be normally distributed. The 0% proportion condition served as the baseline condition. When the proportion of floor data increased (e.g., 5–25%), scores increasingly stacked on the lower end and the data distribution further shifted to the left (or more right skewed).

**Table 4:** Floor proportions with different floor thresholds.

**Proportions of floor data (%)**	**Floor thresholds**	**Mean (*SD*)**	**Score range**
0	No floor	0.86 (1.60)	(−4.62, 5.60)
5	−1.50	1.04 (1.47)	(−1.50, 5.61)
10	−0.95	1.08 (1.41)	(−0.95, 5.61)
15	−0.60	1.12 (1.35)	(−0.60, 5.61)
20	−0.25	1.18 (1.28)	(−0.25, 5.61)
25	0.03	1.25 (1.21)	(0.03, 5.61)

### Data analysis

The simulated data were analyzed using three comparative methods as described previously. While the regular multilevel regression ignore the floor effects, robust standard error approach and multilevel Tobit regression model focuses on the floor effects, with the former one partially addresses the floor effects and the later one fully addresses the floor effects.

### Simulation results

The results are summarized across all 3,000 replications with respect to different methods in dealing with the increasing proportions of floor data in the outcome variables. Results of the relative bias in parameter estimates and standard errors for the three methods (i.e., regular multilevel regression, robust standard error approach, and multilevel Tobit regression model) are presented in Tables 5, 6, respectively. Relative bias in parameter estimates was given as $\left(\hat{\theta }-\theta \right)/\theta$ where $\hat{\theta }$ represented the average estimate and θ was the corresponding population value. Similarly, relative bias in standard error estimates was given as $\left(\hat{\sigma }-\sigma \right)/\sigma$ where $\hat{\sigma }$ represented the average standard error estimate and σ was the corresponding population value. The differences in coverage values, statistical powers, type I error rates, and model fit statistics (i.e., CFI, RMSEA, and SRMR) were negligible across the three methods. Next, the relative biased in parameter estimates and the corresponding standard error estimates were discussed for the three different methods, namely, (1) the ML-based regular multilevel analysis without addressing the floor effects, (2) the robust standard error approach with partially addressing the floor effects (as correction for the non-normality in the floor data), and (3) the multilevel Tobit regression approach with fully addressing the floor effects in data.

**Table 5:** Relative bias in parameter estimates comparing three comparative methods.

**Proportions of floor data (%)**	**Parameter estimates**					
	**γ_10_**			**γ_01_**		
	**Multilevel model floors ignored**	**Robust standard error**	**Multilevel model floors considered**	**Multilevel model floors ignored**	**Robust standard error**	**Multilevel model floors considered**
0	−0.00	−0.00	−0.00	−0.01	−0.01	−0.01
5	−0.05	−0.05	0.00	−0.05	−0.05	0.00
10	−0.10	−0.10	0.00	−0.10	−0.10	0.00
15	−0.15	−0.15	0.00	−0.14	−0.14	0.00
20	−0.20	−0.20	0.00	−0.20	−0.20	0.00
25	−0.26	−0.26	0.00	−0.26	−0.26	0.00

**Table 6:** Relative bias in standard error estimates comparing three comparative methods.

**Proportions of floor data (%)**	**Standard error estimates**					
	**SEγ_10_**			**SEγ_01_**		
	**Multilevel model floors ignored**	**Robust standard error**	**Multilevel model floors considered**	**Multilevel model floors ignored**	**Robust standard error**	**Multilevel model floors considered**
0	0.00	−0.01	−0.01	−0.03	−0.04	−0.04
5	−0.07	−0.09	−0.03	−0.06	−0.09	−0.03
10	−0.14	−0.14	−0.05	−0.10	−0.44	0.00
15	−0.16	−0.13	−0.03	−0.16	−0.18	0.01
20	−0.21	−0.15	−0.02	−0.21	−0.23	−0.01
25	−0.27	−0.17	−0.02	−0.27	−0.27	−0.01

Table 5 presents the relative bias in parameter estimates comparing the three methods. The regular multilevel analysis without addressing floor effects led to the underestimation in parameter estimates and the underestimation became substantial as the proportion of floor data increased. The robust standard error approach which only partially addressing the floor data yielded similar results as the regular multilevel analysis. The reason was that the robust standard error approach only corrected for standard error estimates rather than parameter estimates when the normality assumption was violated. As shown in Table 5, only the multilevel Tobit regression approach yielded the unbiased parameter estimates (i.e., $\left(\hat{\theta }-\theta \right)/\theta$ = 0). This approach fully addressed the floor effects by treating the outcome variable as a left-censored variable. In this case, parameter estimates recovered well in the multilevel Tobit regression approach regardless the proportion of the floor data.

Table 6 summarizes the relative bias in standard errors using the three different methods. There was a clear pattern showing a systematic underestimation of standard errors when floor effects were ignored in regular multilevel analysis. The biases in the robust standard error approach were either similar or smaller than the ones in the regular multilevel analysis approach, and the standard errors were persistently underestimated. Furthermore, as the proportion of floor data increased, the biases tended to be larger. As shown in Table 6, among the three methods, the multilevel Tobit regression approach yielded the smallest bias. Given that most values were around 0.01 and the pattern was stable regardless the proportions of floor data, the degree of underestimation in the standard error estimates for the multilevel Tobit regression approach was negligible. This simulation demonstrated the importance of fully addressing the floor effects in multilevel data and the advantage of using the multilevel Tobit regression over the other methods when analyzing potential floor effects in the data.

## Discussion

This study highlighted the impact of floor effects when the PPVT-4 and the EVT-2 used with a culturally and linguistically diverse population of preschoolers by examining the impact of floor effects in data analysis, including in estimating parameters, the corresponding standard errors, and the tests of significance. Influences of floor effects in multilevel data analysis were investigated through an empirical example and a Monte Carlo simulation study.

Our findings suggest that some caution is warranted when interpreting findings from both PPVT-4 or the EVT-2, especially when these two tests are used with culturally, ethnically and linguistically and ethnical diverse groups. Given the standardized PPVT-4 and EVT-2 are both normed based on the predominantly White, middle-class, English-speaking American samples (Qi et al., 2003), ethnically, culturally and linguistically diverse groups may perform inferiorly relative to the normed sample especially among non-English or non-standard English speaking children. Samples in which 15% or more score at or near to the lowest level in the instruments measurement range may indicate the potential existence of the floor effects which may impact analyses when using traditional analytic methods (Lim et al., 2015). Given the culturally, linguistically and ethnically diverse populations have been shown to perform poorly on the PPVT-4 and the EVT-2 (e.g., Champion et al., 2003; Gonzalez et al., 2015), researchers should attend to with the potential problem of floor effects when using these measures. With increasing populations of language-minority populations (e.g., ELLs, DLLs), considering floor effects in measures warrants close attention.

In this study, it was demonstrated that when analyzing data shown to have floor effects, analytical methods insufficiently addressing the floor effects can lead to misleading results and interpretations First, when investigating the shared-reading intervention effects with a sample of Mexican-American preschoolers enrolled in the randomized clinical study using the PPVT-4 and EVT-2, failing to consider the impact of floor effects led to non-significant effects and quite possibly, misleadingly underestimated the impact of the intervention. Outcomes in this study supported previous findings suggesting that (Hessling et al., 2004), floor effects may undermine the true effects of an intervention, especially among linguistically and culturally diverse populations.

Furthermore, results from the simulation study showed that ignoring floor effects resulted in substantial bias in both parameter estimates and standard errors estimates, and erroneous significance tests. These findings are important and support previous research. McBee (2010) stated conventional statistical methods (e.g., ANOVA, linear regression) produced biased estimates when floor effects were present. Wang et al. (2008) also pointed out the consequence of biased parameter estimates due to the ceiling effects or floor effects. In our study, the two insufficient approaches, namely, the regular multilevel regression ignoring floor effects and the robust standard error approach which only partially addressing floor effects, produced the same parameter estimates. However, the robust standard error approach produced less but still biased standard error estimates due to the “robust” correction. Multilevel Tobit regression was the only method that recovered all the parameter and standard error estimates very well. The multilevel Tobit regression model treated the outcome variables with floor effects as left-censored variables. In other words, scores on the very low end that could not be accurately measured due to the restricted range of the standardized assessments were treated as being left-censored. The Monte Carlo study showed that the multilevel Tobit regression effectively handled floor data. For example, even as low as only 5% of floor data could lead to biased results if floor effects were not adequately and fully addressed. Parameter estimates and standard error estimates were underestimated. The magnitude of the bias became larger as the proportion of floor data increased. Taken together, researchers should consider using the multilevel Tobit regression model to analyze the data with potential floor effects.

Finally, in order to examine floor data, graphs (e.g., histograms) can be easily and effectively used to illustrate whether a substantial proportion of scores stack at the lower end of the distribution. If there is a large percentage of very low scores in their data, researchers should consider the presence of floor effects. Again, as demonstrated in both empirical example and simulation studies, it is important to fully address the floor effects with adequate method, the Tobit regression given that insufficiently addressing the floor effects can result in biased parameter estimates and standard errors, which in turn, can lead to incorrect statistical inferences.

In summary, researchers need to be aware and cautious of the potential for floor effects when analyzing data from ethnically, culturally and linguistically diverse children accessed by the PPVT-4 and the EVT-2. A potential indicator for floor data is the disproportional representation of scores at the lower end of the distribution of the measured scores. Ignoring floor effects can lead to biased parameter estimates and standard errors, and quite possibly serious misleading inferences. It is thereby important for applied researchers who use standardized vocabulary tests with diverse populations to examine their data for floor effects and consider alternatives to the traditional data analysis methods which without fully addressing the floor effects. For modeling outcome variables with floor data, multilevel Tobit regression model is the recommended method for analyzing this type of data.

## Author contributions

LZ initiated the design of the study and presented the work in the 2016 American Psychological Association Annual Convention. LZ and JG wrote the paper. Research data are taken from JG's Project Words of Oral Reading and Language Development (WORLD) efficacy studies (R350A110638: 2011-2014). LZ performed the Monte Carlo modeling.

### Conflict of interest statement

The authors declare that the research was conducted in the absence of any commercial or financial relationships that could be construed as a potential conflict of interest.

## Appendix A

Input Specifications in Mplus for the Multilevel Tobit Regression

Model

TITLE: Syntax for a multilevel tobit model

DATA: FILE IS Floor.dat;

VARIABLE: NAMES are sid tid x1-x7 evt1 evt2 evts1 evts2 x8-

x14 ppvt1 ppvt2 ppvts1 ppvts2;

USEVARIABLES are x1-x7 evts1 evts2 x8-x14;

!outcome variable (ects2) with floor effects

CENSORED ARE evts2 (b);

CLUSTER = tid;

WITHIN = x1-x7 evts1;

BETWEEN = x8-x14;

MISSING is all (9999);

ANALYSIS: TYPE = twolevel;

!robust standard error

ESTIMATOR = MLR;

MODEL: %WITHIN%

evts2 ON x1-x7 evts1;

%BETWEEN%

evts2 ON x8-x14;

OUTPUT: TECH1 standardized

## References

[B1] Baraldi A. N. Enders C. K. (2010). Missing data methods, in The Oxford Handbook of Quantitative Methods, ed Little T. (New York, NY: Oxford University Press), 635–664.

[B2] Browne M. W. (1984). Asymptotically distribution-free methods for the analysis of covariance structures. Brit. J. Math. Stat. Psychol. 37, 62–83. 10.1111/j.2044-8317.1984.tb00789.x 6733054

[B3] Brown T. A. (2006). Confirmatory Factor Analysis for Applied Research. New York, NY: Guilford.

[B4] Bucik N. Bucik V. (2003). Peabody slikovni besedni test (Peabody Picture Vocabulary Test, PPVT-III): Njegove merske lastnosti in uporabna vrednost. = Peabody Picture Vocabulary Test (PPVT-III): psychometric properties and significance for application. Horiz. Psychol. 12, 91–108. Retrieved from: http://www.dlib.si/details/URN:NBN:SI:DOC-QM60VCCW

[B5] Buly M. R. (2005). Leaving no American Indian/Alaska Native behind: identifying reading strengths and needs. J. Am. Ind. Educ. 44, 28–52.

[B6] Capovilla F. C. Capovilla A. G. S. (1997). Desenvolvimento lingüístico na criança dos dois aos seis anos: tradução e estandardização do Peabody Picture Vocabulary Test de Dunn & Dunn. E Da Language Development Survey de Rescorla. Ciência Cognitiva Teoria Pesquisa e Aplicação 1, 353–380.

[B7] Catts H. W. Petscher Y. Schatschneider C. Bridges M. S. Mendoza K. (2009). Floor effects associated with universal screening and their impact on the early identification of reading disabilities. J. Learn. Disabil. 42, 163–176. 10.1177/0022219408326219 19098274 PMC4308976

[B8] Champion T. B. Hyter Y. D. McCabe A. Bland-Stewart L. M. (2003). “A matter of vocabulary” performances of low-income African American Head Start children on the Peabody Picture Vocabulary Test—III. Commun. Disord. Q. 24, 121–127. 10.1177/15257401030240030301

[B9] Cox D. R. Oakes D. (1984). Analysis of Survival Data. London: Chapman and Hall.

[B10] DeAvila E. A. Duncan S. E. (2000). PreLAS2000: English and Spanish Technical Notes. Monterey, CA: CTB/McGraw-Hill.

[B11] Dunn L. M. Dunn D. M. (2007). Peabody Picture Vocabulary Test, 4th Edn. Minneapolis, MN: NCS Pearson.

[B12] Finney S. J. DiStefano C. (2006). Nonnormal and categorical data in structural equation models, in Structural Equation Modeling: A Second Course, eds Hancock G. R. Mueller R. O. (Greenwich, CT: Information Age), 269–314.

[B13] Gonzalez J. Pollard-Durodola S. Saenz L. Soares D. Davis H. Resendez N. (2015). Spanish and English early literacy profiles of preschool Latino English language learner children. Early Educ. Dev. 27, 513–531. 10.1080/10409289.2015.1077038

[B14] Haitana T. Pitama S. Rucklidge J. J. (2010). Cultural biases in the peabody picture vocabulary test-III: testing tamariki in a New Zealand sample. J. Psychol. 39, 24–34.

[B15] Hessling R. M. Schmidt T. J. Traxel N. M. (2004). Floor effect, in Encyclopedia of Social Science Research Methods, eds Lewis-Beck M. S. Bryman A. Liao T. F. T. (Thousand Oaks, CA: SAGE Publications, Inc.), 390–391.

[B16] Horton-Ikard R. Ellis Weismer S. (2007). A preliminary examination of vocabulary and word learning in African American toddlers from middle and low socioeconomic status homes. Am. J. Speech Lang. Pathol. 16, 381–392. 10.1044/1058-0360(2007/041) 17971497

[B17] Hox J. J. Maas C. J. Brinkhuis M. J. (2010). The effect of estimation method and sample size in multilevel structural equation modeling. Stat. Neerl. 64, 157–170. 10.1111/j.1467-9574.2009.00445.x

[B18] Iachina M. Iachina N. (2012). Measuring reliable change of emotional and behavioural problems in children. Psychiatry Res. 200, 867–871. 10.1016/j.psychres.2012.06.023 22789839

[B19] Ji C. Yao D. Chen W. Li M. Zhao M. (2014). Adaptive behavior in Chinese children with Williams syndrome. BMC Pediatr. 14:90. 10.1186/1471-2431-14-90 24708693 PMC3994205

[B20] Keeley J. Keeley T. English J. Irons A. M. (2013). Investigating halo and ceiling effects in student evaluations of instruction. Educ. Psychol. Meas. 73, 440–457. 10.1177/0013164412475300

[B21] Kim Y. Chang H. Lim S. Bak H. (1995). Geurim Eohyuryeok Geomsa [Picture Vocabulary Test]. Seoul: Seoul Community Rehabilitation Center.

[B22] King G. Roberts M. E. (2015). How robust standard errors expose methodological problems they do not fix, and what to do about it. Polit. Anal. 23, 159–179. 10.1093/pan/mpu015

[B23] Klein J. P. Moeschberger M. L. (1997). Survival Analysis: Techniques for Censored and Truncated Data. New York, NY: Springer 10.1007/978-1-4757-2728-9

[B24] Kleinbaum D. G. Klein M. (2005). Survival Analysis: A Self-Learning Text. New York, NY: Springer-Verlag.

[B25] Laing S. P. Kamhi A. (2003). Alternative assessment of language and literacy in culturally and linguistically diverse populations. Lang. Speech Hear. Serv. Sch. 34, 44–55. 10.1044/0161-1461(2003/005) 27764486

[B26] Lim C. R. Harris K. Dawson J. Beard D. J. Fitzpatrick R. Price A. J. (2015). Floor and ceiling effects in the OHS: an analysis of the NHS PROMs data set. BMJ Open 5:e007765. 10.1136/bmjopen-2015-007765 26216152 PMC4521553

[B27] Long J. S. (1997). Regression Models for Categorical and Limited Dependent Variables: Advanced Quantitative Techniques in the Social Sciences. Thousand Oaks, CA: Sage Publications.

[B28] Lonigan C. J. Farver J. M. Nakamoto J. Eppe S. (2013). Developmental trajectories of preschool early literacy skills: a comparison of language-minority and monolingual-English children. Dev. Psychol. 13, 1–15. 10.1037/a0031408 23316767

[B29] Maas C. J. Hox J. J. (2004). Robustness issues in multilevel regression analysis. Stat. Neerl. 58, 127–137.

[B30] McBee M. (2010). Modeling outcomes with floor or ceiling effects: an introduction to the tobit model. Gifted Child Q. 54, 314–320. 10.1177/0016986210379095

[B31] McCabe A. Champion T. B. (2010). A matter of vocabulary II: low-income African American children's performance on the expressive vocabulary test. Commun. Disord. Q. 31, 162–169. 10.1177/1525740109344218

[B32] Muthén B. (1989). Tobit factor analysis. Brit. J. Math. Stat. Psychol. 42, 241–250.

[B33] Muthén B. (1990). Means and Covariance Structure Analysis of Hierarchical Data. UCLA Statistics Series. Los Angeles, CA: Department of Statistics Ucla.

[B34] Muthén B. Asparouhov T. (2010). Beyond multilevel regression modeling: multilevel analysis in a general latent variable framework, in Handbook of Advanced Multilevel Analysis, eds Hox J. Roberts J. K. (New York, NY: Taylor and Francis), 15–40.

[B35] Muthén L. K. Muthén B. O. (1998-2015). *Mplus User's Guide 7th Edn* Los Angeles, CA: Muthén & Muthén.

[B36] Nevitt J. Hancock G. R. (2001). Performance of bootstrapping approaches to model test statistics and parameter standard error estimation in structural equation modeling. Struct. Equat. Model. 8, 353–377. 10.1207/S15328007SEM0803_2

[B37] Pae H. K. Greenberg D. Morris R. D. (2012). Construct validity and measurement invariance of the Peabody Picture Vocabulary Test – III Form A. Lang. Assess. Q. 9, 152–171. 10.1080/15434303.2011.613504 22639554 PMC3358798

[B38] Pakendorf C. Alant E. (1997). Culturally valid assessment tools: Northern Sotho translation of the Peabody Picture Vocabulary Test – Revised. South Afr. J. Commun. Disord. 44, 3–12. 9819964

[B39] Pollard-Durodola S. D. Gonzalez J. E. Saenz L. Soares D. Resendez N. Kwok O. (2016). The effects of content-related shared book reading on the language development of preschool dual language learners. Early Child. Res. Q. 36, 106–121. 10.1016/j.ecresq.2015.12.004

[B40] Proust-Lima C. Dartigues H. Dartigues H. (2011). Misuse of the linear mixed model when evaluating risk factors of cognitive decline. Am. J. Epidemiol. 174, 1077–1088. 10.1093/aje/kwr243 21965187 PMC3551607

[B41] Qi C. H. Kaiser A. P. Milan S. E. Yzquierdo Z. Hancock T. B. (2003). The performance of low-income, African American children on the Preschool Language Scale−3. J. Speech Lang. Hear. Res. 46, 576–590. 10.1044/1092-4388(2003/046) 14696987

[B42] Raudenbush S. W. Bryk A. S. (2002). Hierarchical Linear Models: Applications and Data Analysis Methods, 2nd Edition. Newbury Park, CA: Sage.

[B43] Restrepo M. A. Schwanenflugel P. J. Blake J. Neuharth-Pritchett S. Cramer S. E. Ruston H. P. (2006). Performance on the PPVT–III and the EVT: applicability of the measures with African American and European American preschool children. Lang. Speech Hear. Serv. Sch. 37, 17–27. 10.1044/0161-1461(2006/003) 16615746

[B44] Rock D. A. Stenner A. J. (2005). Assessment issues in the testing of children at school entry. Fut. Child. 15, 15–34. 10.1353/foc.2005.0009 16130539

[B45] Stockman I. J. (2000). The new Peabody Picture Vocabulary Test-III: an illusion of unbiased assessment? Lang. Speech Hear. Serv. Schools 31, 340–353. 10.1044/0161-1461.3104.340 27764472

[B46] Terry N. P. Mills M. T. Bingham G. E. Mansour S. Marencin N. (2013). Oral narrative performance of African American prekindergartners who speak nonmainstream American English. Lang. Speech Hear. Serv. Sch. 44, 291–305. 10.1044/0161-1461(2013/12-0037) 23843654

[B47] Theriault-Whalen C. M. Dunn L. M. (1993). Echelle de Vocabulaire en Images. Richmond Hill, ON: Peabody, Psyscan Corporation.

[B48] Thernstrom A. (2002). The racial gap in academic achievement, in Beyond the Color Line: New Perspectives on Race and Ethnicity in America, 1st Edn., eds Thernstrom A. Thernstrom S. (Stanford CA: Hoover Institution Press), 259–276.

[B49] Thomas-Tate S. Washington J. Craig H. Packard M. (2006). Performance of African American preschool and kindergarten students on the expressive vocabulary test. Lang. Speech Hear. Serv. Schools 37, 143–149. 10.1044/0161-1461(2006/016) 16646217

[B50] Tobin J. (1958). Estimation of relationships for limited dependent variables. Econometrica 26, 24–36. 10.2307/1907382

[B51] Twisk J. Rijmen F. (2009). Longitudinal tobit regression: a new approach to analyze outcome variables with floor or ceiling effects. J. Clin. Epidemiol. 62, 953–958. 10.1016/j.jclinepi.2008.10.003 19211221

[B52] Ueno K. Utsuo T. Iinaga K. (1991). PVT Kaiga Goi Hattatsu Kensa [PVT Picture Vocabulary Development Test]. Tokyo: Chiba Test Center.

[B53] Wang L. J. Zhang Z. Y. McArdle J. J. Salthouse T. A. (2008). Investigating ceiling effects in longitudinal data analysis. Multivar. Behav. Res. 43, 476–496. 10.1080/00273170802285941 19924269 PMC2778494

[B54] Washington J. Craig H. K. (1992). Performances of low-income African American preschool and kindergarten children on the Peabody Picture Vocabulary Test–revised. Lang. Speech Hear. Serv. Sch. 23, 329–333. 10.1044/0161-1461.2304.329

[B55] Washington J. Craig H. K. (1999). Performances of at-risk, African American preschoolers on the Peabody Picture Vocabulary Test–III. Lang. Speech Hear. Serv. Sch. 30, 75–82. 10.1044/0161-1461.3001.75 27764292

[B56] Whitaker S. (2005). The use of the WISC-III and the WAIS-III with people with a learning disability: three concerns. Clin. Psychol. Forum 50, 37–40.

[B57] Whitaker S. (2008). Intellectual disability: a concept in need of revision. Br. J. Dev. Disabil. 54, 3–9. 10.1179/096979508799103350

[B58] Whitaker S. (2010). Error in the estimation of intellectual ability in the low range using the WISC-IV and WAISIII. Pers. Individ. Dif. 48, 517–521. 10.1016/j.paid.2009.11.017

[B59] Whitaker S. (2012). Review of the WAIS-IV - the measurement of low IQ with the WAIS-IV: a critical review. Clin. Psychol. Forum 45–48.

[B60] Whitaker S. Gordon S. (2012). Floor effects on the WISC-IV. Int. J. Dev. Disabil. 58, 111–119. 10.1179/2047387711Y.0000000012

[B61] Williams K. T. (2007). Expressive Vocabulary Test, 2nd Edn. Circle Pines, MN: AGS Publishing.

[B62] Winship C. Mare R. D. (1984). Regression models with ordinal variables. Am. Sociol. Rev. 49, 512–525. 10.2307/2095465

[B63] Yuan K. H. Bentler P. M. (2000). Three likelihood-based methods for mean and covariance structure analysis with nonnormal missing data, in Sociological Methodology, eds Sobel M. E. Becker M. P. (Washington, DC: ASA), 165–200.

[B64] Yuan K.-H. Bentler P. M. Zhang W. (2005). The effect of skewness and kurtosis on mean and covariance structure analysis: the univariate case and its multivariate implication. Sociol. Methods Res. 34, 249–258. 10.1177/0049124105280200

